# Implementation of an organized colorectal cancer screening program through quantitative fecal immunochemical test followed by colonoscopy in an urban low-income community: Guidance and strategies

**DOI:** 10.1016/j.clinsp.2023.100278

**Published:** 2023-08-26

**Authors:** Ulysses Ribeiro, Adriana Vaz Safatle-Ribeiro, Maurício Sorbello, Poliana Helena Rosolem Kishi, Rejane Mattar, Vera Lucia Pagliusi Castilho, Elenice Messias Do Nascimento Goncalves, Fábio Kawaguti, Carlos Frederico Sparapan Marques, Venâncio Avancini Ferreira Alves, Sérgio Carlos Nahas, José Eluf-Neto

**Affiliations:** a Departments of Gastroenterology, Universidade de São Paulo, São Paulo, SP, Brazil; b Fundação Oncocentro de São Paulo (FOSP), São Paulo, SP, Brazil; c Instituto do Câncer do Estado de São Paulo, Hospital das Clínicas, Faculdade de Medicina, Universidade de São Paulo (ICESP-HCFMUSP), São Paulo, SP, Brazil; d Preventive Medicine, Universidade de São Paulo, São Paulo, SP, Brazil; e Pathology, Universidade de São Paulo, São Paulo, SP, Brazil

**Keywords:** Screening, Colonoscopy, Fecal immunochemical test, Colorectal cancer, Adenoma, Polyps

## Abstract

Fecal Immunochemical Test (FIT) followed by a colonoscopy is an efficacious strategy to improve the adenoma detection rate and Colorectal Cancer (CRC). There is no organized national screening program for CRC in Brazil. The aim of this research was to describe the implementation of an organized screening program for CRC through FIT followed by colonoscopy, in an urban low-income community of São Paulo city. The endpoints of the study were: FIT participation rate, FIT positivity rate, colonoscopy compliance rate, Positive Predictive Values (PPV) for adenoma and CRC, and the rate of complications. From May 2016 to October 2019, asymptomatic individuals, 50–75 years old, received a free kit to perform the FIT. Positive FIT (≥ 50 ng/mL) individuals were referred to colonoscopy. 10,057 individuals returned the stool sample for analysis, of which (98.2%) 9,881 were valid. Women represented 64.8% of the participants. 55.3% of individuals did not complete elementary school. Positive FIT was 7.8% (776/9881). The colonoscopy compliance rate was 68.9% (535/776). There were no major colonoscopy complications. Adenoma were detected in 63.2% (332/525) of individuals. Advanced adenomatous lesions were found in 31.4% (165/525). CRC was diagnosed in 5.9% (31/525), characterized as adenocarcinoma: *in situ* in 3.2% (1/31), intramucosal in 29% (9/31), and invasive in 67.7% (21/31). Endoscopic treatment with curative intent for CRC was performed in 45.2% (14/31) of the cases. Therefore, in an urban low-income community, an organized CRC screening using FIT followed by colonoscopy ensued a high participation rate, and high predictive positive value for both, adenoma and CRC.

## Highlights

• This study describes the implementation of an organized colorectal cancer (CRC) screening in an urban low-income community.
• Quantitative fecal immunochemical test (FIT) followed by colonoscopy is an efficacious strategy to improve the adenoma detection rate and CRC.
• FIT followed by colonoscopy ensued a high participation rate, and high predictive positive value for adenoma and CRC.

## Introduction

Colorectal Cancer (CRC) is the second leading cause of cancer-related death worldwide with an increasing incidence in younger patients resulting in a great impact in the quality of life.^1^^,^^2^

In Brazil, according to the National Cancer Institute (INCA), CRC is the second most frequent tumor among men and women, after prostate cancer and breast cancer, respectively. Almost 46,000 new cases of CRC are expected in Brazil for each year of the 2023‒2025 triennium. The values correspond to an estimated risk of 20.78 new cases per 100,000 men and 21.41 per 100,000 women.^3^ There is a wide variation in incidence per 100.000 persons among different Brazilian regions, from < 10 in the North and Northeast, while in the South and Southeast, the rate is around 30 in men and 20 in women.^3^

CRC develops, in most cases, from premalignant adenomatous polyps, according to the adenoma-carcinoma sequence.^4^^,^^5^ and offers a window of opportunity for screening. In average-risk individuals, screening through colonoscopy and resection of adenomas reduces the incidence and mortality of CRC.^6^, ^7^, ^8^, ^9^

Early treatment of superficial lesions (adenomatous polyps and/or early CRC) through endoscopic resection can be less harmful than surgical resection. The survival rate varies according to the stage at the time of the diagnosis, accounting for 90% when the tumor is still located in the intestinal wall, 68% when the disease affects lymph nodes, and only 10% when the disease is metastatic.^6^^,^^10^^,^^11^

Several tests for CRC screening are available, including Fecal Occult Blood Test (FOBT) (guaiac), Fecal Immunochemical Test (FIT), sigmoidoscopy, colonoscopy, CT Colonography (CTC), fecal DNA testing, and capsule endoscopy.^12^, ^13^, ^14^, ^15^ FIT followed by colonoscopy represents the preferred methods of CRC screening worldwide. A quantitative FIT has high specificity, and colonoscopy can diagnose and provide therapeutic management of superficial lesions.^9^^,^^16^, ^17^, ^18^, ^19^, ^20^, ^21^

Despite the long-established recommendations (Brazil, 2010), there is no organized nationwide population screening program for CRC in Brazil. There is minimal reporting of the results of the FOBT or FIT as an initial tool for CRC screening, except for a few pilot regional studies.^22^, ^23^, ^24^, ^25^

In order to achieve the potential benefits of CRC screening, all steps of the screening must be optimized, including identification and outreach to the target population, high performance (high accuracy and specificity) and availability of screening test, high-quality colonoscopy, treatment, surveillance and aftercare guidelines.^26^^,^^27^ Upon diagnosis of lesions, adequate treatment should be given to the patients, including surgical resection and adjuvant therapy.^14^^,^^28^^,^^29^

Thus, the main aim of this research was to describe the implementation of an organized screening program for CRC through quantitative FIT followed by colonoscopy in positive FIT individuals, in an urban low-income community of São Paulo city under the public Healthcare System domain (Brazilian Public Healthcare System). The endpoints of the study were: FIT participation rate, FIT positivity rate, colonoscopy compliance rate, colonoscopy diagnosis, positive predictive values for adenoma and CRC, and the rate of major complications.

## Material and methods

From May 2016 to October 2019, an organized CRC screening program was implemented in an urban low-income community in the east zone of the city of São Paulo, Brazil. The project was developed by the Departments of Gastroenterology, Preventive Medicine, and Pathology of Faculdade de Medicina, Universidade de São Paulo (FMUSP), in association with Fundação Oncocentro de São Paulo (FOSP) and Hospital Santa Marcelina (HSM), the latter responsible for managing all the involved Basic Health Units (BHU). All institutions are public or philanthropic with a partnership of the São Paulo State Public Health Secretary. More than 500 professionals including community health agents, nurses, secretaries, technicians, physicians, statisticians, and computer technicians were involved in the project, some of them voluntary. The institutional Review Board approved the study and informed consent was obtained from all participants (Number = 51237114.8.0000.0065).

Individuals at average risk received a free kit to perform the FIT (Flaconet Fecal Test from Eiken Chemical, Japan; a ColOff feces collector from Coloff Industrial Ltda-EPP, Brazil; and a brochure with instructions for use). Individuals with greater or equal to 50 ng/mL or 10 µg/g of FIT value were considered positive and were contacted and referred for colonoscopy.

### Inclusion criteria

Asymptomatic individuals of both sexes, from 50 to 75 years of age who live in an urban low-income community of East Zone of São Paulo city were invited to participate.

### Exclusion criteria

- Age under 49 or over 76 years.
- Previous diagnosis of inflammatory bowel disease, or Hereditary Colorectal Cancer Syndromes, for instance, Familial Adenomatous Polyposis and Lynch Syndrome.
- Personal history of CRC.
- Previous colorectal surgical resection.
- No signed informed consent form.

A questionnaire was completed by the selected individuals with questions regarding address, sex, age, race, schooling, personal history of colonoscopy, personal history of polyps and family history of CRC.

### Screening program organization

The project included 13 BHUs under HSM management, that selected the target population to be invited to receive the FIT. The screening program was free for all participants.

In order to implement the screening program, it was necessary to make changes in the routine healthcare procedures. BHU developed a campaign to raise awareness of the need to undergo screening for CRC, clarifying benefits, risks, results, colonoscopy procedures and possible treatment. Participants with a positive FIT who missed colonoscopy appointments received a home visit from a community health agent or nurse to know the reason for non-attendance and to provide them another opportunity to undergo the procedure. Reminders, phone calls, and other strategies were used to increase compliance.

Step by step screening project is described in Table 1.

**Table 1:** Step-by-step description of the CRC screening project.

**Step A ‒ Preparation**	Acquisition of consumer and permanent items
	Selection of eligible individuals, scheduling them by each BHU
	Hiring a team of technicians to analyze the samples
	Team training for field work and data analysis
**Step B ‒ Enrolling participants to carry out the FIT**	Information about the CRC screening program and signature of the Informed Consent form
	Completion of the questionnaire
	Delivery of the FIT kit, and scheduling the date for returning the FIT at BHU
**Step C ‒ FIT Assessment**	Quantitative analysis of FIT
	Notification of the FIT result to BHU and contacting the patients with FIT results by community health agent
	Education of the patients by the community health agent about positive results and the next steps
**Step D ‒ Colonoscopy**	Scheduling for positive FIT patients in HCFMUSP
	Diagnostic colonoscopy with biopsies, and endoscopic resections of lesions smaller than 2 cm and without suspicion of CRC
	Individuals with suspicious CRC or lesions greater than 2 cm were referred to ICESP-HC-FMUSP for endoscopic submucosal dissection, surgical resection, and/or adjuvant therapy
	Histopathological analysis of all specimens
	Colonoscopy follow-up of individuals undergoing endoscopic resection
	Family counseling in cases diagnosed with CRC
**Step E – Management of advanced CRC**	Scheduling the first institutional consultation at ICESP-HCFMUSP
	Staging, clinical and surgical oncological evaluation
	Definition of the multidisciplinary therapeutic plan
	Treatment according to the therapeutic plan
	Clinical and surgical follow-up
**Step F ‒ Data Analysis**	Assessment of colonoscopy and pathological findings related to the FIT-positive result
	Descriptive evaluation of colonoscopy findings and pathological aspects of CRC-related lesions

CRC, colorectal cancer; BHU, basic health units; FIT, fecal immunochemical Test; ICESP-HCFMUSP, Instituto do Cancer do Estado de São Paulo, Hospital das Clínicas, Faculdade de Medicina da Universidade de São Paulo.

### Delivery of kits and collection of stool samples

FIT Kit was delivered by the community health agent or nurse practitioner, to the individuals’ homes. One fecal sample test was used in this study. A positive FIT cut-off value was set in 50 ng/mL, or 10 µg/g, and the participants with an equal result or above this level were referred for colonoscopy.

Some volunteers or health-related professionals were also involved in the distribution and collection of kits. Delivery of the kits, in this case, allowed an additional opportunity for counseling, transmitting information about the program, and for providing instructions about using the FIT.

### Colonoscopy

Patients with positive FIT were notified and scheduled for a colonoscopy exam. There was a thorough explanation of the procedure, including risks and benefits, expected results, and preparation to be carried out prior to the exam. Colonoscopies were performed by senior endoscopists, who had performed at least 3000 colonoscopies. High-definition magnified flexible video colonoscopes were used in the study (Fujinon, series 590 or 600).

Bowel preparation was done with fiber restricted diet, cathartics, and mannitol, which was graded by the endoscopist, according to the Boston Bowel Preparation Scale.^29^ A complete colonoscopy was defined when the colonoscope reached the cecum or terminal ileum.

The exams were performed in the Colonoscopy Unit of the Colorectal Surgical Division, Department of Gastroenterology at HCFMUSP. There was a specific schedule for screening colonoscopies, and the interval between the FIT result and diagnostic colonoscopy was less than three months. When polyps and small superficial lesions were detected, polypectomies and endoscopic mucosal resections were performed at the time of the diagnosis. Lesions greater than 2 cm or malignant neoplasia were referred to the Therapeutic Endoscopic Unit at ICESP-HCFMUSP, for endoscopic resection or directly to the surgical team.

If an adenoma or serrated polyp was diagnosed, a follow-up surveillance colonoscopy was scheduled based on recommendations of the US Multi-Society Task Force on Colorectal Cancer.^19^

### Histopathological study

Specimens obtained by endoscopic and/or surgical resection were fixed in 10% formalin solution and were sent for histopathological analysis. This evaluation was performed by the Pathology Department at ICESP-HCFMUSP or IC-HCFMUSP.

For the categorization of precursor lesions and CRC, the revised Vienna classification was used,^30^ and for Serrated Lesions (LS), the Organization's digestive system tumor classification World Health Organization, 2019 (WHO, 2019) was utilized.

Advanced Adenoma (AA) was defined as: a lesion greater than 10 mm or with a villous component or high-grade Dysplasia (DAG); Early CRC: the maximum extent of invasion restricted to the submucosal layer; Advanced CRC: lesion extends beyond the submucosal layer.^31^ The advanced neoplasm was defined as AA or CRC.

TNM Classification System from Union International Cancer Control was utilized for CRC staging.

### Post-colonoscopy referral

Colonoscopy exam reports were sent to BHU and to the individuals, as well as post-exam guidance.

### Data management

The variables were collected prospectively and entered into a RED CAP database. There was strict management and data monitoring, especially for individuals with a positive FIT. There was an early warning system for positive FIT, and the after-treatment service followed evidence-based guidelines and was offered to all patients detected with cancer or pre-invasive lesions at the time of evaluation.

There was a continuous quality assurance program aimed at the identification and correction of possible failures.^18^^,^^29^^,^^32^

### Statistical analysis

Data analysis was performed using SPSS software, version 26.0 (SPSS Inc., Chicago, IL). Descriptive statistics included frequencies with percentages for nominal variables and mean with Standard Deviation (SD) for continuous variables. The authors evaluated the differences between the groups using Chi-Square tests (Pearson) for nominal variables and t-test for continuous variables. Positive Predictive Value (PPV) of colonoscopy findings were calculated. All *p*-values were two-sided and a value < 0.05 was considered statistically significant.

## Results

**Fig. 1:**
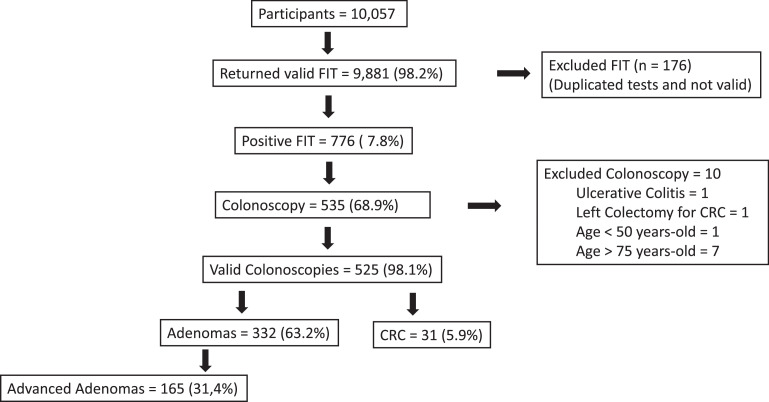
Study flowchart demonstrating the main findings of an organized colorectal cancer screening through Fit followed by colonoscopy. (FIT, Fecal Immunochemical Test; CRC, Colorectal Cancer).

This study showed that it is possible to implement an organized CRC screening program in a low-income community with the integration of many health professionals. The results that are described below corroborated the effectiveness of this program. During the study period, 10,057 individuals returned the stool sample for analysis, of which 9,981 (98.2%) were valid. Quantitative analysis was done per single round, after obtaining a single stool sample (Fig. 1).

Demographic data was obtained through participant surveys and can be seen in Table 2. Women represented 64.8% (6398/9881) of the individuals. The age group of 50 and 59 years old corresponded to 45.1% (4,453/9,881) of participants. Race was indicated by 9712 individuals and the majority were white (46.7%; 4,617/9,712). Education level was informed in 9,312 individuals, and 55.3% (5179/9312) had not completed elementary school.

**Table 2:** Demographics of the entire screened population (*n =* 9881) for adenomatous lesions and colorectal cancer.

Age		60.7 (SD = 6.6)	(%)
	50‒59	4453	45.1
	60‒69	4254	43.1
	70‒75	1174	11.9
Sex			
	Female	6398	64.8
	Male	3483	35.2
Race			
	White	4617	46.7
	Yellow	82	0.8
	Indigenous	8	0.1
	African–American	853	8.6
	Brown	4151	42
	Unknown	169	1.7
Schooling			
	Elementary incomplete	5179	55.3
	Elementary complete	1364	14.6
	High school incomplete	541	5.8
	High school complete	1582	16.9
	Full higher education	615	6.6
	Unknown	87	0.6
Colonoscopy before			
	Yes	1362	13.9
	No	8450	86.1
Polyps before			
	Yes	200	2.2
	No	9095	97.8
Family history of colorectal polyps			
	Yes	312	3.2
	No	9335	94.5
	Unknown	234	2.4
Family history of colorectal cancer			
	Yes	328	3.5
	No	9080	96.5
FIT	> 50ng/mL	776	7.8
	< 50ng/mL	9105	92.1

SD, standard deviation; FIT, fecal immunochemical test.

Among participants, 3.2% had a family history of colorectal polyps, and 3.5% had a family history of CRC.

The positive FIT rate was 7.8% (776/9,881). The mean positive FIT value was 720.3 ng/mL, the median was 164 ng/mL, with a standard deviation of 4424 ng/dL (95% CI 50‒9,999).

Of the 776 individuals scheduled for colonoscopy, 535 attended and underwent the procedure, corresponding to a 68.9% (535/776) compliance rate. Ten of the participants did not fulfill the inclusion criteria and were excluded: outside the age group (*n =* 8), previous CRC surgical resection (*n =* 1), and previous diagnosis of inflammatory bowel disease (*n =* 1). Thus, 525 individuals underwent colonoscopy analysis.

### Descriptive analysis of colonoscopy findings

Complete colonoscopy was achieved in 99.4% (522/525). There were two incomplete exams due to stenosing neoplasms, and one due to loop formation.

There were no early or late major colonoscopy complications. Bleeding after resection occurred in 1.5% (8/525) of individuals and were treated endoscopically.

Potential bleeding lesions not related to CRC or adenomatous lesions, such as orifical, vascular, and inflammatory findings were identified in 117 individuals. Normal colonoscopy accounted for 8.8% (46/525) of cases.

Adenoma detection rate was 63.2% (332/525), and adenoma per colonoscopy rate was 1.7 (0‒14).

Advanced adenomatous lesions were diagnosed in 31.4% (165/525) of individuals of which 15.8% (26/165) had high-grade dysplasia.

Most of the adenomatous polyps 55.3% (494/894) were in the right colon, while 44.7% (400/889) were in the left colon, including 13.2% (53/400) in the rectum.

CRC was diagnosed in 5.9% (31/525) of individuals, defined as adenocarcinoma *in situ* in 3.2% (1/31), intramucosal adenocarcinoma in 29% (9/31), and invasive adenocarcinoma in 67.7% (21/31). Synchronous adenocarcinomas were detected in 6.4% (2/31) (Fig. 1).

Endoscopic potentially curative CRC treatment was performed in 45.2% (14/31) of the cases. Seventeen individuals underwent surgical resection.

Prevalence and PPV for adenomatous lesions, advanced adenomatous lesions, and CRC are presented in Table 3.

**Table 3:** Histophathological final results after colonoscopy of 525 screened individuals.

Colonoscopic findings	Number	Histophathological diagnosis	Number	PPV
Adenomatous Polyps	332	Adenoma	332	63.2%
		Advanced adenoma	165	31.4
		Serrated lesion	27	5.1
Tumors	31		31	5.9
		*In situ*	1	
		Intramucoso	9	
		Invasive	21	4

PPV, positive predictive value; 95% CI, 95% confidence interval.

## Discussion

This is the first organized CRC screening program using quantitative FIT followed by colonoscopy in a specific target population in Brazil. The study demonstrated the feasibility of the implementation of this program in an urban low-income population of a developing country.

In order to implement an organized CRC screening, several steps had to be addressed. After choosing a target population to be screened, it was essential to educate all health professionals about the importance of prevention. These health agents informed the target population about the likelihood of decreasing the risk of developing CRC after adenomatous polyp resection. All involved BHU were responsible for signing the informed consent, delivering, and receiving the returned kit. Community health agents and nurses helped to deliver the FIT and explain how it was to be used. After informing the individuals of their positive FIT, a colonoscopy was recommended for diagnostic biopsies and possible endoscopic treatment. Adequate treatment was crucial, especially for those individuals diagnosed with advanced adenoma or CRC. Most importantly, close oversight and quality control of all processes and evaluation of the entire program was done and programmatic changes were implemented, if necessary.^26^^,^^27^

The most challenging step of the program was convincing the population of the benefits of screening, even when asymptomatic.^9^^,^^27^ The authors solved this problem by educating the population about CRC. The commitment of engaged health professionals was essential for the success of the program.

Fortunately, CRC screening programs are expanding around the world. The shift from gFOBT to FIT has led to increased adherence to CRC screening. FIT requires only a stool sample, with no need for dietary or drug restrictions.^12^^,^^21^ Additionally, quantitative tests have high sensitivity and specificity, leading to more consistent results compared to gFOBT.^7^^,^^9^^,^^16^^,^^17^^,^^33^

As implemented in many developed regions of the world, for instance, Europe, Asia, USA, Canada, and also in some Latin American countries, including Uruguay and Chile, FIT followed by colonoscopy represents an efficacious strategy to improve the detection rate of adenoma and CRC lesions. Positive FIT significantly increased the colonoscopy yield for cancer and advanced adenoma.^9^^,^^19^^,^^27^^,^^31^^,^^34^

It was shown that overall pooled sensitivity of FITs for CRC was 0.79 (95% CI 0.69 to 0.86), and specificity of 0.94 (95% CI 0.92 to 0.95). FIT overall accuracy was (95% CI 93% to 97%).^9^^,^^20^

FIT PPV for cancer ranged from 2.9% to 7.8% and for advanced adenoma ranged from 33.9% to 63.2%.^9^^,^^28^ In this study, FIT PPV for CRC was 5.9%, for adenomas was 63.2%, and for advanced adenomas was 31.4% (Table 3).

Depending on the FIT cut-off level, both the sensitivity and specificity vary. Sensitivity decreased with increasing cut-off values, from 0.86 (95% CI 0.75 to 0.92) at cut-off values less than 20 µg/g to 0.67 (95% CI 0.59 to 0.74) at cut-off values greater than 50 µg/g. FIT cut-off values less than 20 µg/g might have the best combination of sensitivity and specificity for CRC (89% and 91%, respectively). The optimal cut-off level should be determined according to colonoscopy availability. Therefore, adequate resources are an important consideration when choosing a cut-off threshold, especially in developing countries with limited healthcare infrastructure.^15^^,^^17^^,^^34^ In this organized study, a cut-off value was 50 ng/mL (10 µg/g), leading to FIT positive rate of 7.8%. This cut-off value ensured that an adequate number of appointments were available for colonoscopies for the target population.

Systematic reviews, cohort studies, prospective observational studies, case-control studies, and long-term follow-up studies of patients after polypectomy have demonstrated the reduction of CRC incidence and mortality in individuals who underwent colonoscopy screening.^6^^,^^9^^,^^19^^,^^35^, ^36^, ^37^, ^38^, ^39^ Conversely, there are some potential harms caused by colonoscopy screening including unnecessary anxiety and morbidity, needless economic costs, and exposure to the risk from invasive diagnostic procedures, and lesion resections.^40^^,^^41^

As recommended by US Multi-Society Task Force on CRC statement, FIT completion rate for those offered testing should be greater than 60%; the proportion of returned FIT that cannot be processed by lab of < 5%; the colonoscopy compliance rate for those with a positive FIT should be around 80%, and the detection rate of ADR > 45% in men and > 35% in women.^9^^,^^19^^,^^33^

In the current study, FIT completion rate was above 96%. The colonoscopy compliance rate was 69%, less than the Task Force suggestion of 80%. However, some studies from other countries have also shown low compliance rate for colonoscopy, some of them below 40%.^42^, ^43^, ^44^, ^45^ Colonoscopy compliance rate from this study may be explained by the difficulty of patients traveling to the Hospital das Clínicas to undergo colonoscopy, since the hospital is located in a central area of the city, far from the individual's home; a lack of finances to travel to the hospital; and the inability of patients to find someone to escort them home after the colonoscopy. On the other hand, in-patient hospital day clinics for bowel preparation improved adherence.

The analysis of colonoscopy findings in this study showed the high quality of the performed exams, in accordance with the recommendations of the most relevant world endoscopic societies guidelines.^9^^,^^19^^,^^29^^,^^32^ Adenomas were detected in 63.2%, and high-grade dysplasia and CRC were diagnosed in 10.7% (56/525) of the individuals. All patients had their treatment done at the Institution and have been followed after treatment. These rates are similar to other published studies.

Most of the studied individuals (86%) did not have the opportunity to undergo a colonoscopy before this screening project.

Similarly, to other studies, almost two-thirds of the individuals were female. This might be explained by the high rate of screening for other diseases in females including breast and uterine tumors and their willingness to be examined. Most of the male population are employed and could not interrupt work to have a colonoscopy, as well as being afraid of receiving a colonoscopy.^9^^,^^20^

Implementing and sustaining a screening program, such as CRC screening, in a middle-income country like Brazil presents unique challenges and opportunities. It requires not only extensive human resources but also a robust health system capacity.^46^ One of the barriers to the implementation of a CRC screening program in a middle-income country is the allocation of sufficient resources. Limited funding and competing health priorities can hinder the establishment and maintenance of a comprehensive screening program. Additionally, the availability and accessibility of screening facilities, trained healthcare professionals, and diagnostic services may pose significant challenges, particularly in remote or underserved areas. There are also several facilitators that can support the successful implementation of a CRC screening program. Awareness campaigns and education initiatives targeting both the general population and healthcare providers play a crucial role in increasing acceptance and participation rates. Collaboration and partnerships between government agencies, non-governmental organizations, and healthcare institutions can help mobilize resources, share expertise, and enhance program sustainability. Furthermore, leveraging existing infrastructure, such as primary care networks, can improve the integration of screening services into routine healthcare delivery. Moreover, involvement, enthusiasm, and commitment were the main factors for the success of this study.

There are some limitations of this research. First, a colonoscopy was performed in a hospital far from the individuals' homes, which probably decreased the rate of colonoscopy compliance. If this program is used in São Paulo for community screening, geographically expanding the locations for performing colonoscopies would increase the compliance rate. Second, the present findings are limited by the use of a restricted number of FIT samples. Third, continued financial support for the project is necessary for its success and future endurance.

In summary, the major impact of this project was the diagnosis of malignant lesions (high-grade dysplasia and CRC) in 10% of asymptomatic individuals in a low-income, and low-educated population, most of whom never had an opportunity to have a colonoscopy before.

The potential for reducing mortality through cancer screening can only be achieved if people diagnosed with adenoma and/or CRC receive prompt and adequate attention when lesions are detected. All patients in the present study with positive lesions received carefully planned treatments and follow-up care.

## Conclusions

In an urban low-income community, the efforts of more than 500 people allowed the execution of an organized CRC screening that resulted in the immediate treatment of 30.2% of individuals who underwent colonoscopy and presented adenoma and CRC.

FIT as the initial method for screening, followed by colonoscopy ensued a high participation rate, and high PPV for adenoma and CRC.

Therefore, the implementation of an organized screening program with high-quality control was essential to maximize the impact of the colonoscopy. Importantly, it resulted in diagnosing lesions in the early stage, leading to possible cures with a direct impact on quality of life.

## Authors' contributions

U. Ribeiro and J. Eluf-Neto: Proposed and designed the study, analyzed the data, and wrote the manuscript. A.V. Safatle-Ribeiro, M. Sorbello, P.H.R. Kishi, F. Kawaguti, C.F.S. Marques: Collaborated in data collection, enrolled patients, colonoscopy examination and interpretation of the data. V.F.A. Alves: Carried out the pathologic review. R. Mattar, V.L.P. Castilho, E. M.N. Goncalves: Carried out the analysis of FIT collected. S.C. Nahas, U. Ribeiro Jr, A.V. Safatle-Ribeiro: Performed a final critical revision. All authors read and approved the version to be published.

## Disclosures and funding sources

This research was funded by FAPESP/PPSUS Fundação de Amparo à Pesquisa de São Paulo (FAPESP) and DECIT/Programa de Pesquisa para o SUS (PPSUS) under nº 14/50112-2.

FIT (OC-Sensor Diana by Japanese company, Eiken Chemical Co., Tokyo, Japan), and Coloff sanitarium kit were kindly donated to the Project by Eiken company and Coloff Industrial Ltda-EPP.

## Conflicts of interest

The authors declare no conflicts of interest.
